# Recreational activity after cementless total hip arthroplasty in patients older than 75 years

**DOI:** 10.1007/s00402-021-03896-y

**Published:** 2021-05-03

**Authors:** Alexander Zimmerer, Luis Navas, Stefan Kinkel, Stefan Weiss, Matthias Hauschild, Wolfgang Miehlke, Marcus Streit

**Affiliations:** 1 ARCUS Sportklinik, Rastatterstr. 17-19, 75179 Pforzheim, Germany; 2 Department of Orthopedics and Orthopedic Surgery, University Medicine Greifswald, Ferdinand-Sauerbruch-Straße, 17475 Greifswald, Germany

**Keywords:** Sport, Physical activity, Hip replacement, Mid-term, Old patient

## Abstract

**Introduction:**

This retrospective study aimed to compare activity levels before and at mid-term follow-up after primary cementless total hip arthroplasty (THA) in patients older than 75 years.

**Materials and methods:**

A cohort of 79 patients with a mean age at surgery of 78 years (range 76–84 years) was evaluated 6.3 years (range 4–8 years) after cementless THA due to osteoarthritis and was followed up with a questionnaire to determine their activity level. Pre- and post-operative recreational activities were assessed at routine follow-up using the University of California, Los Angeles activity score, and the Schulthess Clinic sports and activity questionnaire. Post-operative health-related quality of life was measured using Veterans Rand 12-item survey (VR-12).

**Results:**

Six years after THA, 72% of preoperatively active patients had returned to activity. Comparing activity preoperatively (before the onset of symptoms) and 6 years after THA, the number of disciplines and session length has decreased significantly. A significant decline in high-impact activities was observed, while participation in low-impact activities significantly increased.

**Conclusion:**

The majority of patients maintained a recreational activity level in the mid-term after primary cementless THA. However, a change in disciplines toward low-impact activities was observed.

## Introduction

Total hip arthroplasty (THA) is one of the most successful orthopedic surgeries with an increasing caseload per year [1, 2]. As life expectancy rises, an increasing number of senior citizens are expected to participate in sports. The majority of patients’ desire to maintain an active lifestyle and engage in sports after THA will increase. For younger patients, a high rate of return-to-activity after THA could be shown [3, 4]. However, data on the activity level after cementless THA in patients older than 75 years are scarce in the literature. To the best of our knowledge, there are only data for short-term follow-up available [5]. Therefore, we conducted this study to analyze the return-to-activity rate and to assess the physical and recreational activity of patients older than 75 years undergoing primary cementless THA at mid-term follow-up. The hypothesis of our study was that the majority of patients treated by cementless THA would be able to return to regular recreational activity.

## Methods

### Patient selection

**Fig. 1:**
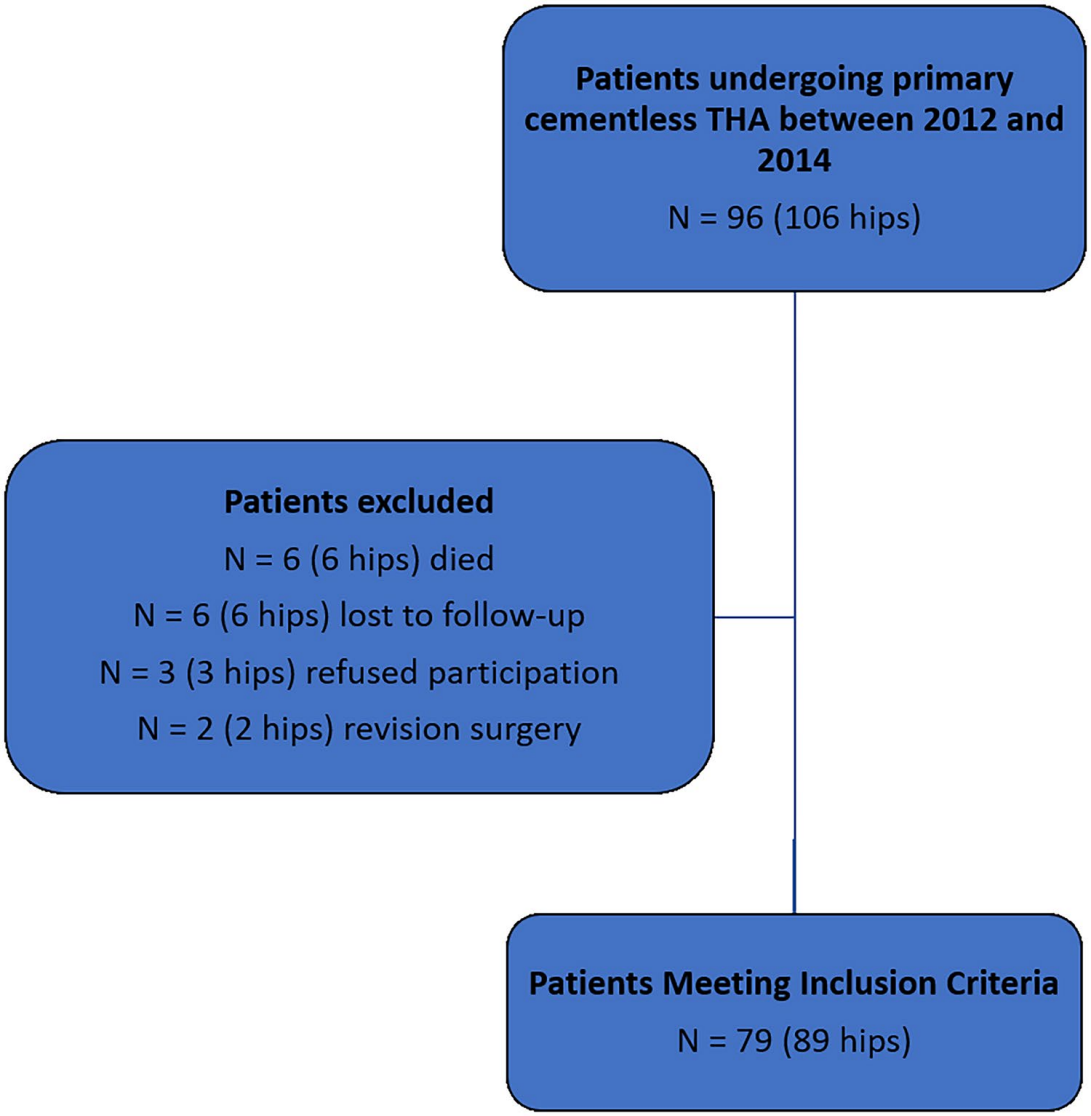
Flowchart illustrating the number of patients excluded from the study, lost to follow-up, and those who met inclusion criteria

The present retrospective study comprises a cohort of 96 patients older than 75 years following primary cementless THA performed in a multi surgeon series (6 surgeons) between January 2012 and December 2014 at our institution (Fig. 1). Exclusion criteria were primary cemented or hybrid THA, revision surgery, and age at surgery of 74 years or younger. Six patients had died at a mean follow-up of 6.3 years (range 5–8), three patients refused to participate, and six patients were lost to follow-up. The THA failed in two patients during the study period and were therefore excluded from the study (one aseptic stem loosening, one septic revision), leaving 79 patients who were asked to complete the Schulthess Clinic Activity Score [6] and the Veterans Rand 12-Item Health Survey (VR-12) Physical Component Score (PCS) and Mental Component Score (MCS) [7]. The Schulthess Clinic Activity Score determines the athletic ability before the beginning of the symptoms with the present condition. In addition, patients’ physical activity was prospectively assessed using the University of California, Los Angeles activity scale (UCLA) [8]. Activities such as skiing or cycling were defined as high-impact sports, while activities such as walking or ergometer training were defined as low-impact sports.

All patients gave informed consent. The ethics commission of the Landesaerztekammer Baden-Wuerttemberg, Germany, approved all procedures (F-2019-006), and the study was conducted following the Helsinki Declaration of 1975, as revised in 2008.

All patients received a cementless cup and a cementless femoral component. A cementless Corail stem (Depuy Orthopaedics, Warsaw, Indiana, USA) was used in all hips. A cementless Allofit acetabular cup (Zimmer, Warsaw, Indiana, USA) was used in 8 hips and a Pinnacle acetabular cup (Depuy Orthopaedics, Warsaw, Indiana, USA) in 81 hips, respectively. Ten patients received bilateral THA. A minimal-invasive posterior approach was used in 28 hips, a transgluteal Bauer approach in 40 patients, and a direct anterior approach (DAA) in 21 patients. Recommendations for return to activities following THA met the consensus guidelines from members of the Hip Society and the American Association of Hip and Knee Surgeons [9].

### Statistics

Descriptive statistics for all continuous variables were reported as means (rang). Differences between preoperative and post-operative data were examined with a *t* test and Wilcoxon signed-rank test. Categorical variables were reported using count and percentage. McNemar test was conducted to detect differences. A post hoc power analysis resulted in a power of 0.85 with an alpha error of 0.05 and an effect size of 0.3 due to the given sample size. Statistical analyses were conducted using SPSS statistical software (IBM SPSS Statistics for Windows, version 26.0.0; IBM Corp).

## Results

### Demographics

A total of 79 patients (89 hips) met inclusion criteria and were included in the analysis (Fig. 1). The mean age was 78 (75–84) years, the mean body mass index (BMI) was 26.6 (20.2–41.8) kg/m^2^, and the mean follow-up was 6.3 (4–8) years. Surgery was performed in 33 men and 46 women. The diagnoses leading to arthroplasty were primary osteoarthrosis in 81 and secondary osteoarthrosis due to congenital dysplasia of the hip in 8 hips. Forty-six THAs were performed on the right side, 43 on the left side (Table 1).

**Table 1:** Patient demographic data

	Value
Total no. of patients	79 (89 hips)
Laterality, *n* (%)	
Right	46 (52)
Left	43 (48)
Sex, *n* (%)	
Male	33 (42)
Female	46 (58)
Age, year	78 (75–84)
Body mass index, kg/m^2^	26.6 (20.2–41.8)
Follow-up time, year	6.3 (5–8)

Values are shown as n (%), respectively, as the mean ± SD (range)

### Recreational activities

**Fig. 2:**
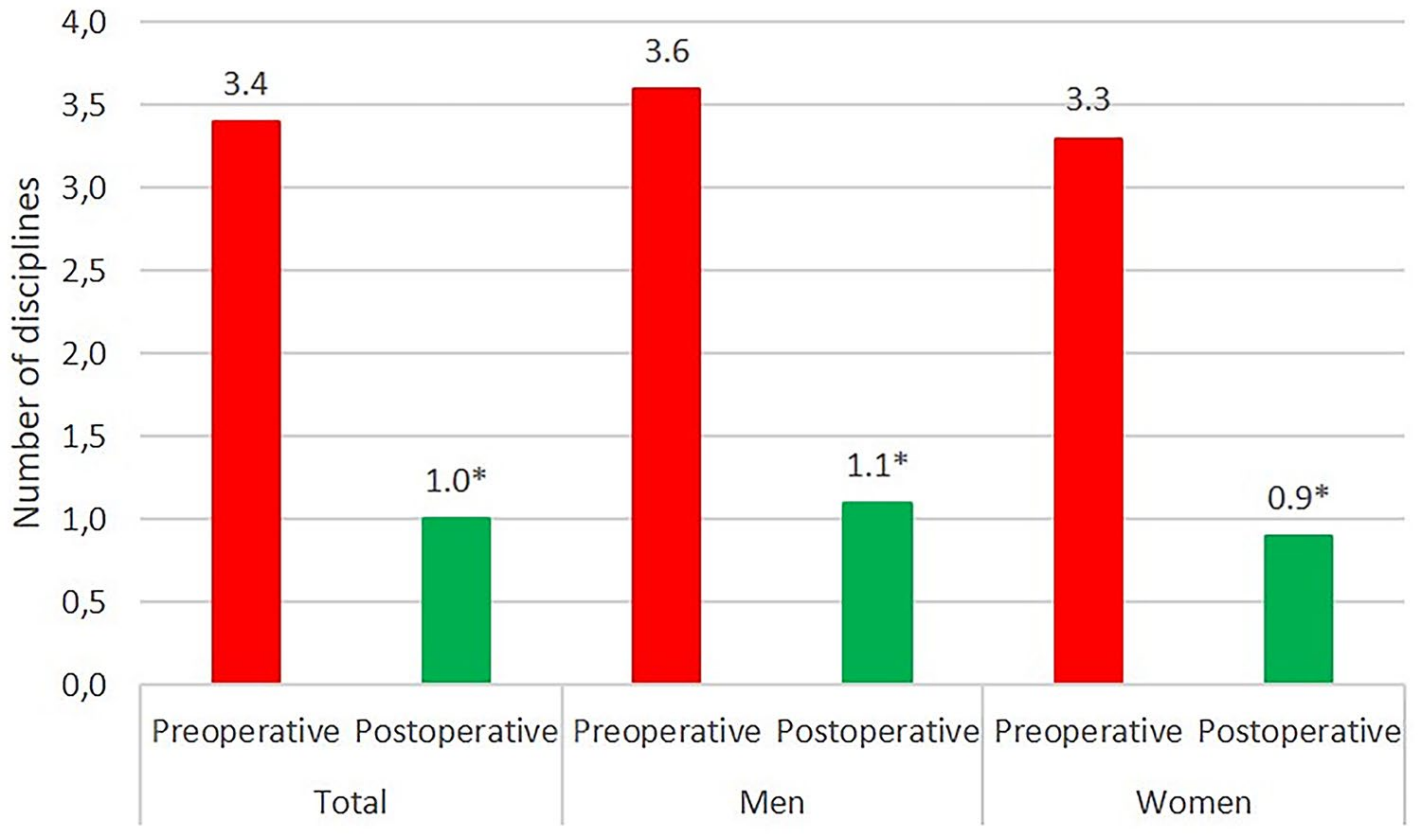
Number of sport disciplines patients participated in before the onset of the first symptoms and after cementless THA. The number of sport disciplines decreased significantly. Asterisks indicate significance (*p* < 0.0001)

For a total of 79 patients, a complete questionnaire regarding recreational activity was available. After surgery, 56 of 79 patients (71%) were active in at least one recreational activity, compared with 78 of 79 (99%) preoperatively, giving a return-to-activity rate of 72%. None of the patients who had been inactive before surgery took up new sporting and recreational activities postoperatively. Patients performed an average of one different sport disciplines at the last follow-up, which differed significantly from the number before the onset of the first symptoms (3.4 disciplines; *p* < 0.0001). This significant decline was confirmed in the individual consideration of men and women, respectively (Fig. 2). Concerning single disciplines, outdoor activities such as hiking and biking revealed a significant decrease (*p* < 0.0001). In contrast, short walks increased significantly (*p* < 0.001) (Tables 2 and 3). Seventy-two percent of the patients returned to recreational activities within 1 month after surgery, and 26% resumed sporting activities within 3 months.

**Table 2:** Sports disciplines patients participated in before and after cementless total hip arthroplasty^a^

Sports discipline	Numbers of patients participating before surgery	Numbers of patients participating after surgery	Difference (%)
Fitness training	58	20	− 75^b^
Short walks	2	16	+ 700^b^
Long walks	62	13	− 79^b^
Ergometer training	8	11	+ 38
Swimming	40	11	− 73^b^
Biking	61	9	− 85^b^
Gymnastics	2	4	+ 100
Hiking	40	0	− 100^b^
Nordic walking	17	0	− 100^b^

^a^Several disciplines demonstrated significant decrease, which was mainly observed in outdoor sports. There was a significantly increase in short walks

^b^Significant (*p* < 0.0001)

**Table 3:** Top sports disciplines of different patient groups before and after cementless total hip arthroplasty^a^

	Preoperative participation (% of patients)	Postoperative participation (% of patients)	Difference (%)
Top sports in men			
Long walks	78	16	− 79^b^
Biking	78	13	− 83^b^
Fitness training	72	28	− 61^b^
Swimming	47	16	− 66^c^
Short walks	3	25	+ 833^b^
Top sports in women			
Long walks	77	0	− 100^b^
Biking	75	10	− 87^b^
Fitness training	73	23	− 68^b^
Swimming	52	8	− 85^b^
Hiking	50	0	− 100^b^
Short walks	2	17	+ 750^b^

^a^Several disciplines demonstrated significant decrease, which was mainly observed in outdoor sports. There was a significantly increase in short walks in male and female patients

^b^Significant (*p* < 0.0001)

^c^Significant (*p* = 0.002)

### Extent of activities

**Fig. 3:**
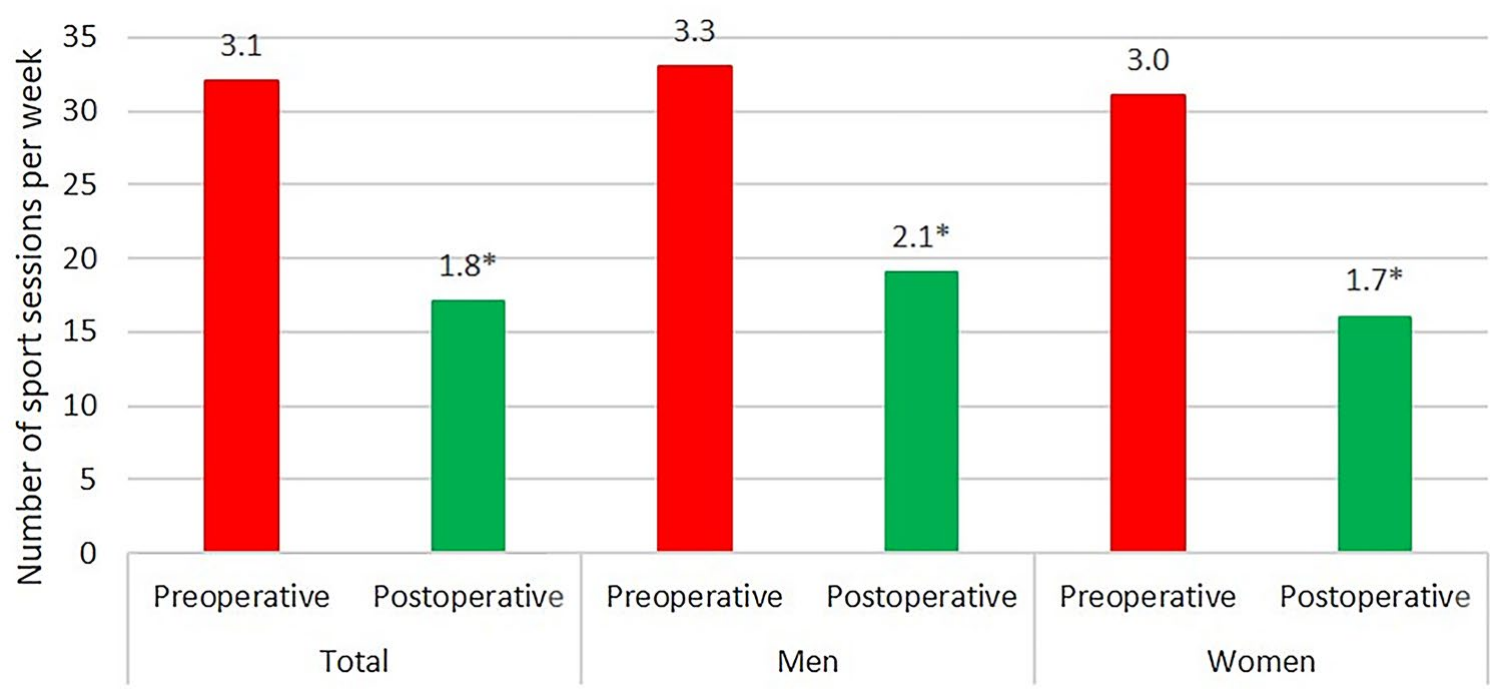
Number of sports sessions patients participated in before the onset of the first symptoms and THA. The average number of sports sessions decreased significantly after cementless THA implantation. Asterisks indicates significance (*p* < 0.0001)

The sports frequency (sessions per week) decreased significantly from the level before the onset of the first symptoms to the current state: patients were active 3.1 times before the onset of the first symptoms and 1.8 times postoperatively per week, respectively (*p* < 0.0001). When analyzing the subgroups, male patients participated more often in sports than female patients at the last follow-up, though this difference was not significant (2.1 vs. 1.7 times per week, respectively, *p* = 0.354). (Fig. 3).

**Fig. 4:**
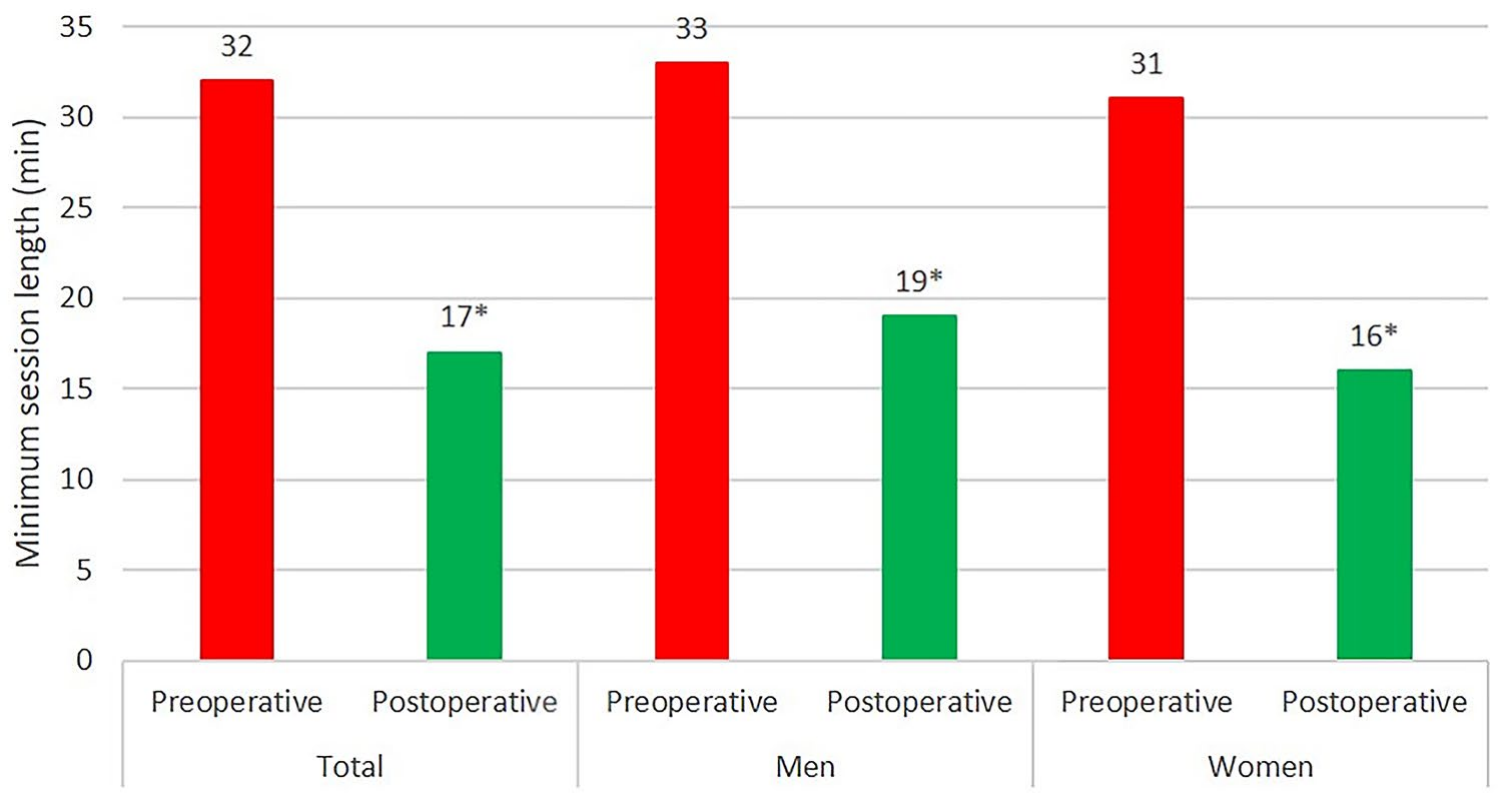
Minimum length of each sport session before the onset of the first symptoms and after cementless THA. Asterisks indicate significance (*p* < 0.0001)

The overall minimum session length decreased from 32 min before surgery to 17 min at the last follow-up (*p* < 0.0001). In addition, minimum session length decreased in male and female patients (Fig. 4).

As a reason for less physical activity, 55% stated that they were less physically capable, 40% were more anxious, and 14% indicated feeling insecure. Eighty-nine percent of the patients reported an improvement in sporting and recreational activity due to the THA.

### Analysis of pre- versus postoperative patient-reported outcome score measurements

Analysis of preoperative and at follow-up reported UCLA Score demonstrated statistically significant improvement from 3.3 (1–8) preoperatively to 3.7 (1–6) at last follow-up (*p* < 0.007). None of the patients were highly active with a UCLA Score ≥ 7. The post-operative rating of the VR-12 PCS was 43.5 (18.5–56.6) and MCS 41.8 (30.8–52.3) points.

## Discussion

Life expectancy is rising in general, so that it can be assumed that an increasing proportion of the population will need a THA. Besides, it can be assumed that the proportion of pensioners who engage in sporting activities will increase. However, data on the activity level after cementless THA in patients older than 75 years are scarce in the literature.

The present study's main findings are that a large majority of patients could return to any manner of recreational behavior after cementless THA. We could demonstrate a return-to-activity rate of 72% at the latest follow-up (6.2 years). As stated before, there are little data available for this age cohort. Ortmaier et al. reported a return-to-activity rate of 92% after cementless short-stem THA [5]. In their study, three age groups (< 60, 60–70, and > 70) were analyzed, whereby the return-to-activity rate was not differentiated between the individual age groups. Considering the overall return-to-sport rate in the literature, a decrease in sports activity was also reported for younger patients after THA [10, 11].

Looking at the level of physical activity in more detail, there was a shift from high-impact to low-impact activities. The most popular activities after surgery were short walks and indoor activities. These activities were in line with the Hip Society members’ consensus guidelines and the American Association of Hip and Knee Surgeons [9]. The decrease in high-impact activity is consistent with the results described in the literature [5, 12, 13].

In total, the frequency and the session length changed significantly post-operative for both men and women. The activity frequency and the session length after surgery significantly decreased for male and female patients. Ortmaier et al. were able to show that the older group displayed a lower frequency and duration of recreational activity [5]. When patients were asked for the reasons for the change in their activities, they reported a high level of anxiety and reduced physical resilience. Even though the overall volume and intensity decreased, 89% of the patients stated an improvement in sporting and recreational activity due to the THA compared to the initial situation before surgery. In general, participation in sport declines with age [14, 15]. Cross-sectional studies reported lower sports participation in people with higher age [16–18], and older age groups are less likely to be regularly active [19, 20]. Thereby, women seem to be less likely than men to achieve regular physical activity [21].

In the present study, PROMs were also evaluated to survey sporting activity. The UCLA activity score significantly improved in our patients (3.3–3.7 points), but none of the patients were highly active with a UCLA Score ≥ 7. Ortmaier et al. reported a mean UCLA activity score of 6.2 for patients > 70 years after a mean follow-up of 20 months, which was significantly lower than the younger groups’ scores [5]. The present study's lower UCLA activity score may be because our cohort exhibits a higher average age than Ortmaier’s group (mean age 78 vs. 74 years) and is less active due to age. The VR-12 MCS and PCS were analyzed at the latest follow-up. The achieved scores [PCS 43.5 (18.5–56.6), MCS 41.8 (30.8–52.3)] are comparable to those reported in the literature for the general population of the equivalent age.[22].

There are several limitations of this study. First of all, the study was designed in a retrospective manner, and patients reported on sport and recreational activities in which they might have been active years before. However, this is due to the questionnaire concept, which has been described previously and is commonly accepted. It must also be noted that the time of the first symptoms, as assessed in the questionnaire used, may have been several years ago. Besides, the preoperative VR-12 questionnaire is lacking. Finally, the patients were not clinically examined for the final follow-up, but only with a questionnaire, and there was no radiological examination at the time of follow-up. Another point worth mentioning is the variety of approaches used, which could potentially influence the results. However, the number of individual groups was too small to carry out a dedicated analysis. Our study’s strength is that it provides detailed information on physical activity after cementless THA in patients older than 75 years, a population for which there are scarcely any data available.

## Conclusions

There is a lack of information on activity in patients older than 75 years receiving THA. We could demonstrate that a large proportion returns to recreational activity and maintains the level in the mid-term follow-up. However, there was a significant decrease in the number of sport disciplines, the frequency, and duration of the sports sessions from preoperatively to postoperatively. Most patients were engaged in low-impact activities.
